# An examination of the curvilinear relationship between person-job fit and innovative behavior: the moderating role of abusive supervision in South Korea

**DOI:** 10.3389/fpsyg.2024.1408108

**Published:** 2024-08-30

**Authors:** Jinhee Kim, Soojin Lee

**Affiliations:** ^1^College of Business Administration, Chonnam National University, Gwangju, Republic of Korea; ^2^College of Global Business, Korea University, Sejong-si, Republic of Korea

**Keywords:** person-job fit, innovative behavior, abusive supervision, triphasic model of stress, conservation of resources theory

## Abstract

Although literature suggests that a higher person-job fit leads to more innovative behavior, some recent studies have shown inconsistent results with the assumption of such a linear relationship between the two constructs. Considering these inconsistent findings, the present study aims to examine a curvilinear relationship between person-job fit and innovative behavior. Innovative behavior represents an individual's actions that come up with, realize, and apply novel ideas within the job environment, and person-job fit, which pertains to the value congruence between the job and individual, can be a critical predictor of innovative behavior. Drawing on the triphasic model of stress and the conservation of resources theory, this study hypothesizes that person-job fit has a non-linear relationship with innovative behavior, and that abusive supervision moderates this relationship. The regression analysis results of the 180 employee-supervisor dyadic data revealed that person-job fit and innovative behavior have a non-linear relationship. Furthermore, the non-linear relationship is (1) weakened (linearly positive) when abusive supervision is high and (2) strengthened when abusive supervision is low. By integrating multiple theoretical lenses, the present study offers a more sophisticated understanding of individual employees' psychological reactions to job fit discrepancies and their innovative outcomes in organizational settings. Theoretical and practical implications and directions for future research are also discussed.

## 1 Introduction

Innovative efforts are essential for organizations to survive intense market competition (Zhang et al., 2022). However, such innovative efforts often fail not because of the lack of technology but because of the lack of employees' acceptance and involvement (Clayton, 1997; Kwon and Kim, 2020). Thus, to innovate successfully, it is necessary to facilitate employees' innovative behavior (Van de Ven, 1986; Tajeddini and Trueman, 2008). Innovative behavior, as a crucial resource for innovation, has received significant attention from scholars and practitioners (Huang et al., 2019). Innovative behaviors refer to an individual's actions that come up with, realize, and apply novel ideals to organizational settings with the intent to improve work, the team, or the organization (West and Farr, 1989). While researchers have long explored the antecedents of innovative behavior, one of the constructs that has received scholarly attention was person-job (P-J) fit. P-J fit reflects the match between an individual's ability and the job's demands (i.e., demands-abilities) and that between the individual's desires and the job's attributes (i.e., needs-supplies) (Edwards, 1991). As the concept stresses the value congruence between individuals and jobs (Kristof, 1996), it has a close connection with job outcomes such as innovative behavior (Tang et al., 2021). Given that innovative behavior begins with one's cognition of a problem and one's will to solve it to improve the job context (West and Farr, 1989; Scott and Bruce, 1994), how the individual views his or her jobs may be one of critical keys to the manifestation of innovative behavior. Indeed, the literature has acknowledged that a higher P-J fit can lead to more innovative behavior (e.g., Afsar et al., 2015; Huang et al., 2019; Tang et al., 2021; Alqhaiwi et al., 2023).

However, despite the seemingly obvious predictions and supporting evidence, the original literature, as well as some inconsistent findings, provide insights that the relationship between P-J fit and innovative behavior may not be simply linear. Stress, such as a discrepancy between individual and job characteristics, can stimulate a coping strategy to modify the situation (c.f., person-environment fit theory, French et al., 1974, 1982), and innovative behavior is an effective way of dealing with stress (Janssen, 2004). In line with the notions, Astakhova et al. (2017) found that demand-ability fit, a subdimension of P-J fit, has a concave relationship with risk-taking propensity, which is relevant to innovative behavior that inevitably entails risks (Anderson et al., 2004). Another study found that need-supply and demand-ability fits–both subdimensions of P-J fit–had nonlinear effects on port employees' innovative performance (Jiang et al., 2021). These findings are consistent with Luis et al.'s (2020) finding that occupational stress has a U-shaped relationship with innovative performance under certain conditions. If so, asserting the assumption of a linear relationship between P-J fit and innovative behavior may be somewhat simplistic and may overlook individuals' proactivity.

Drawing from the triphasic model of stress (Selye, 1950, 1974) and the conservation of resource theory (COR theory; Hobfoll, 1989), this study examines the curvilinear relationship between P-J fit and innovative behavior. The triphasic model of stress, often claimed as a father of stress research (Rosch, 1999; Ursin and Eriksen, 2004), suggests that when stress initially arises, performance decreases because individuals do not actively cope with a low level of stress; however, once the stress is heightened, individuals actively engage in actions to solve the situations as their coping strategies are activated (Selye, 1950, 1974). The conservation of resources theory argues that individuals cope with stressful situations not only by preserving their resources but also by investing resources to protect themselves from further losses (Hobfoll, 1989). Linking two theories to explain employees' coping efforts with the lack of P-J fit, the present study suggests that employees with poor P-J fit can also demonstrate innovative behavior driven to take drastic action to reverse disadvantaged situations.

Moreover, little is known about why conflicting insights have been brought about by previous findings. The present study suggests that the pattern of the relationship between P-J fit and innovative behavior may differ depending on the influence of surrounding factors. While jobs are mostly performed through concerted efforts with supervisors, the supervisor's role in innovative behavior is crucial (Basu and Green, 1997). Abusive supervision is a major source of stress that employees face in organizational settings, which causes psychological pain and depletes their resources (Tepper, 2007; Whitman et al., 2014; Qin et al., 2018). Abusive supervision may weaken the non-linear relationship between P-J fit and innovative behavior by discouraging the innovative efforts of vulnerable employees due to fewer resources (i.e., those with poor P-J fit) as well as those undergoing a minor decline in their P-J fit (i.e., those with less than perfect P-J fit). Conversely, the non-linear relationship may be strengthened in the absence or the low level of abusive supervision because employees can feel free to respond to job misfit, as it should be.

This study contributes to the discipline of applied psychology in four ways: first, questioning the previous assumption that a higher P-J fit leads to higher innovative behavior (e.g., Afsar et al., 2015; Huang et al., 2019; Tang et al., 2021; Alqhaiwi et al., 2023), this study suggests that the relationship is not simply linear but can be curvilinear. Much like old saying “the right man in the right place,” the old wisdom about individual attitudes toward job fit and their behavioral outcomes traditionally has emphasized that “fit matters” (Kristof-Brown et al., 2005, p. 316). While this is true, the findings of the present study add that “poor fit also matters” in that the efforts of those grappling with discrepancies in job fit may also lead to innovation. Second, this study adds nuance to our knowledge about the relationship between P-J fit and innovative behavior by suggesting that the pattern of the relationship can significantly differ depending on the influence of contextual factors, which may explain the contradictory insights from previous research. Thus, this study highlights the importance of considering contextual factors in understanding employees' responses to job discrepancies. Third, this study integrated the triphasic model of stress and COR theory to understand employees' coping responses. The present study suggests that multiple theories can connect at their intersection to explain individuals' complex psychological reactions and behavioral outcomes in organizational settings. Finally, the present study underscores the significance of P-J fit as a key personal resource (Wheeler and Halbesleben, 2009) that can mitigate the negative impact of adverse work conditions.

This study tests these hypotheses using a sample of 180 employee-supervisor dyads working in several companies in South Korea. South Korea is often characterized by a high power distance and a performance-oriented culture, and abusive supervision is more frequently observed (Hofstede, 1980; Fukuyama, 1995). In this context, employees are more likely to perceive job-fit discrepancies and supervisor evaluations. Thus, South Korea is a well-suited context to test hypotheses about the curvilinear relationship between P-J fit and innovative behavior and the moderating role of abusive supervision.

## 2 Theoretical background and hypotheses development

### 2.1 Person-job fit and innovative behavior

Innovative behavior is defined as an intentional action to generate, implement, and utilize novel ideas within the workplace with the intent of enhancing job performance, the group, or the organization (West and Farr, 1989). Innovative behavior is an action that inevitably entails uncertainty because it brings something to the organization that had not existed there and thus involves the risk of failure (Janssen, 2004). Furthermore, pushing it through requires substantial effort, as there are a number of hurdles such as the inertia of people going back to old ones or opposition from those afraid of the changes (Afsar et al., 2015; Zhu and Zhang, 2019). Nonetheless, it is the individuals who recognize issues while performing jobs, generate ideas, and engage in such risky, laborious behavior (Scott and Bruce, 1994). Hence, innovative behavior is likely to stem from employees' attitudes toward their jobs (Kwon and Kim, 2020).

One concept that best reflects how individuals look at their jobs is P-J fit. The literature has traditionally acknowledged that a higher P-J fit leads to more positive organizational outcomes (Edwards, 1996; Collins and Amabile, 1999; Kim et al., 2009). When job characteristics and demands align with an individual's abilities and needs, they are likely to respond more creatively to given situations (Kristof-Brown et al., 2005). In addition, individuals' knowledge and abilities may enable them to be more creative in their work by facilitating cognitive thinking processes such as the generation of solutions (Amabile, 1988). On the other hand, low P-J fit has been considered to negatively affect organizational outcomes. For example, when an individual's ability is too low than the job requires, performance is likely to suffer from inefficient work processes (Cable and DeRue, 2002). Conversely, when an individual's ability is too high than the job requires, the one may become complacent. Additionally, it can be predicted that if a job does not give rewards an individual needs, the one is likely to lose interest in the job.

In line with these arguments, several empirical studies on the relationship between P-J fit and innovative behavior have reported supporting results (e.g., Afsar et al., 2015; Huang et al., 2019; Tang et al., 2021; Alqhaiwi et al., 2023). For instance, Afsar et al. (2015) argued that P-J fit can lead to innovative behavior via innovation trust. However, recent research has shown results that seem somewhat inconsistent with those findings. For example, Astakhova et al. (2017) reported that demand-ability fit–one of two P-J fit dimensions–had a U-shaped relationship with risk-taking propensity. They explained that employees whose abilities are too high or low than the job requires can show a higher risk-taking propensity as a strategy to regain a sense of control, which provides insight into the relationship between P-J fit and innovative behavior that has a risk-involving nature. Jiang et al. (2021) also reported that need-supply and demands-abilities fits, two dimensions of P-J fit, had a nonlinear effect on the port workforce's innovative performance. Intriguingly, Luis et al. (2020) found that occupational stress, such as high job responsibility, conditionally had a curvilinear relationship with innovative behavior; to some extent, stress was detrimental, but once it reached a point, it boosted innovative behavior.

### 2.2 The triphasic model of stress and the conservation of resource theory

The present study argues that the relationship between P-J fit and innovative behavior can follow a curvilinear rather than a linear pattern. The non-linear relationship between P-J fit and innovative behavior can be better understood by considering two theories that emphasize individuals' coping responses to stress: the triphasic model of stress (Selye, 1950, 1974) and COR theory (Hobfoll, 1989). The triphasic model of stress, proposed by Selye (1950, 1974), posits that performance initially decreases at a low level of stress (the alarm phase). It is because individuals often make little effort to cope with the low level of stress, which still causes confusion and distractions from their jobs. However, once the level of stress arises (reactance phase), individuals feel compelled to take action to address the heightened stress, and such coping responses can result in increased performance. However, when the stress reaches an excessively high level (exhaustion phase), it renders coping efforts futile, and performance decreases again. Drawing on this logic, Leung et al. (2011) show in their study that role conflict has a U-shaped relationship with innovative performance when perceived support for innovation is low. The main tenet of COR is that individuals strive to acquire, maintain, protect, and increase their resources (Hobfoll, 1989). From the perspective of theory, individuals are mainly motivated by two motives: the conservation motive, which pertains to the individual's efforts to preserve their resources, and the investment motive, which pertains to the individual's devotion of resources to protect against current or potential losses (Qin et al., 2014).

### 2.3 Establishing a conceptual model

#### 2.3.1 The non-linear relationship between person-job fit and innovative behavior

**Figure 1:**
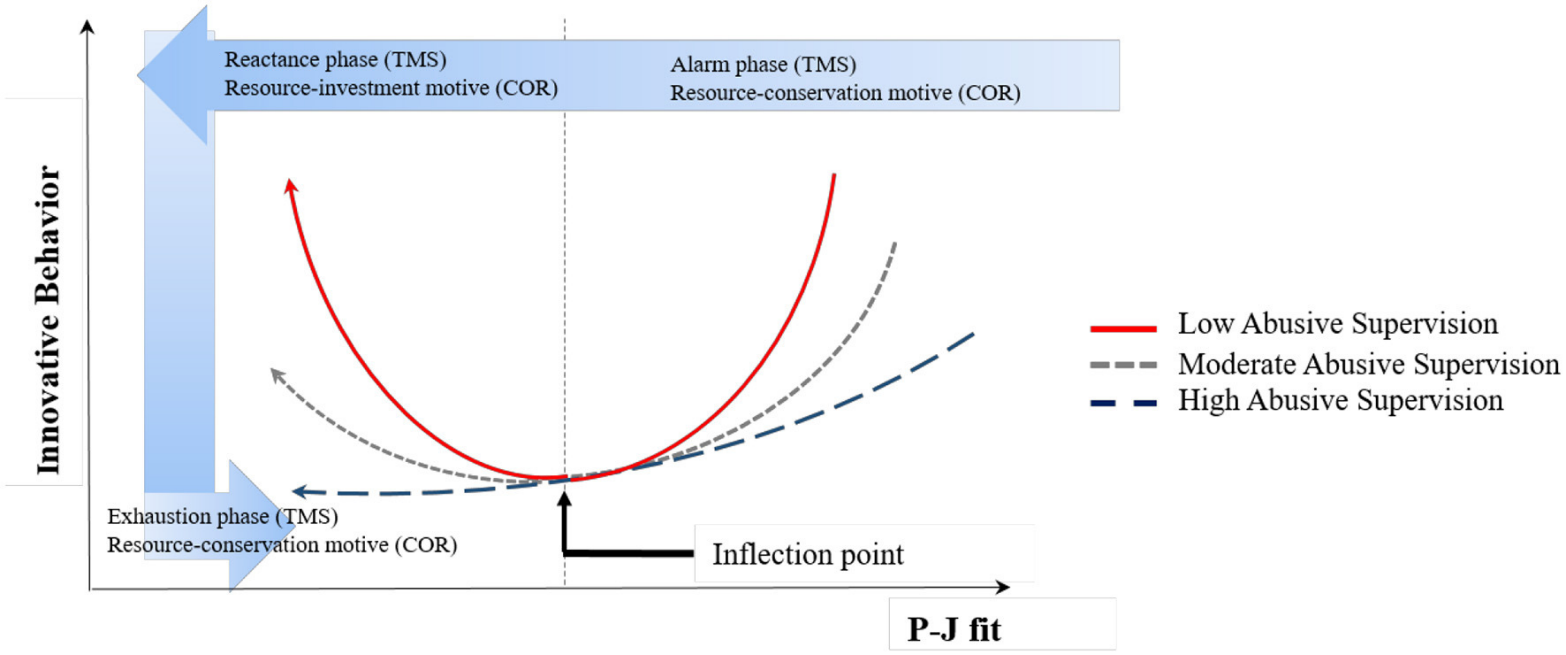
The conceptual model of the relationship between P-J fit and innovative behavior.

Although employees with perfect P-J fit are likely to demonstrate high innovative behavior, innovative behavior will likely diminish when the level of P-J fit initially diminishes but is still higher than or similar to the average level (see Figure 1). From the perspective of the triphasic model (Selye, 1950, 1974; Leung et al., 2011), when the stress occurring from job discrepancies is not significant, individuals may not be aware of it or may not feel compelled to take drastic action to cope with it. However, stress will still decrease innovative behavior, causing confusion and distracting their attention from the job, thus hindering the generation of novel ideas and their willingness to make changes in their jobs. From the perspective of the COR theory, the low level of stress arising from discrepancies will undermine individuals' cognitive resources and, in turn, inhibit their intention to accomplish valuable resource tasks (Luis et al., 2020). However, for small losses, they are unlikely to invest other resources in innovative changes. Given that innovative behavior requires substantial cognitive and emotional resources (Janssen, 2004), the gain (i.e., filling a relatively small gap in one's P-J fit level) by making such a significant investment will not pay off its efforts and risks. Instead, they are likely to attempt to preserve their resources by managing their job performance (Qin et al., 2014).

However, once P-J fit reaches the inflection point and continues to diminish past this point, employees are likely to start to increase innovative actions to address the rising stress stemming from the lack of P-J fit. Individuals not only passively respond to stress, but also willingly take risks of throwing out old patterns that no longer fit the pressing demands to embrace new ones if the situation requires them to do so (Hobfoll et al., 2018). From the perspective of the triphasic model, once the incongruence between an employee and their job is revealed as significant, it becomes an unignorable source of annoyance and anxiety. When elevated stress is perceived as no longer trivial but rather threatening, coping behaviors are activated (Lazarus and Folkman, 1984). To reduce their negative influences, individuals can be motivated to resolve discrepancies by modifying the job context, such as job characteristics, working methods, or procedures (Janssen, 2004). From the perspective of the COR theory point of view, when the loss of resources becomes significant and reaches a point where individuals cannot withstand further losses, a mandate for change may overwhelm their fear (Leung et al., 2011; Zhou et al., 2019). Since gains in resource scarcity are more valuable than those in a resource-abundant situation (Hobfoll et al., 2018), they will be highly motivated to invest their resources in improving of P-J fit. Indeed, not only do they have little things to lose, but the gains (e.g., better fit with P-J fit, improved job outcomes) they may have will exceed the benefits of remaining in the status quo if those innovative efforts are successful.

Taken together, this study postulates that when discrepancies with perfect P-J fit are relatively small, albeit with little stress, individuals will be less engaged in innovative behavior. However, when the discrepancies are revealed as serious (i.e., when P-J fit reaches a threshold), individuals will increasingly be motivated to engage in innovative behavior as a coping behavior to resolve it. Therefore, the present study hypothesizes the following:

*Hypothesis 1. P-J has a non-linear (U-shaped) effect on innovative behavior, such that innovative behavior initially diminishes as the P-J fit diminishes to a point; after this point, innovative behavior starts to increase*.

#### 2.3.2 The moderating role of abusive supervision

Abusive supervision refers to a subordinate's perception of the extent to which a supervisor consistently exhibits hostile actions, both verbally and nonverbally, without physical contact (Tepper, 2000). Abusive supervision is known to be a significant stressor that employees may experience in organizational settings (Tepper, 2007). By experiencing a supervisor's public mockery and inconsiderate and disrespectful behaviors, employees may suffer damage to their self-esteem and have doubts about whether their contribution is respected by the organization and whether their jobs are meaningful enough for their own development (Liu et al., 2012). Employees are also likely to perceive that their supervisors treat them punitively and arbitrarily, making them feel insecure (Rousseau and Aubé, 2018). Thus, abusive supervision generates high levels of stress in employees because they consume enormous cognitive and emotional resources.

When the level of abusive supervision is high, the non-linear effects of P-J fit on innovative behavior should be weakened. From the COR's perspective, engaging in innovative behavior is a significant investment that demands substantial effort (Janssen, 2004). Employees with higher P-J fit should struggle to cope with abusive supervision; thus, they will not be able to invest their resources in such costly behavior. Given the high probability of failure of innovative behavior, they will also be reluctant to engage because of the fear of being punished or the potential negative consequences of failure (Janssen, 2004; Carmeli et al., 2010; Zhu and Zhang, 2019). As a result, the innovative behavior of those with a higher P-J fit may diminish at a high level of abusive supervision. Abusive supervision may even exacerbate the decline in innovative behavior of those with lower P-J fit. The COR theory suggests that people suffering from resource scarcity are more vulnerable to additional losses than those who have rich resources (Hobfoll, 2001). Those with low P-J fit, who already suffer from the heightened stress coming from the discrepancies with their jobs, will likely lack the resources (i.e., congruence with the job) to offset the negative influences of abusive supervision. Furthermore, fear of the supervisor's negative reactions may be greater for those with low P-J fit because they are likely to have received unpleasant attention from their supervisor due to unsatisfactory job outcomes (Cable and DeRue, 2002). Soon, mounting stress will put their reserves in danger of depletion, and as such, they will likely minimize any innovative efforts to defend themselves from further losses (Hobfoll, 2001), which corresponds with the explanation of the triphasic model that too high levels of stress make people cease coping efforts.

In contrast, at a low level of abusive supervision, the detrimental effects of abusive supervision are minimized or, at least, significantly reduced. Such a situation is similar to when an important stressor is controlled. Since there is little or no stressor that diverts employees from their jobs, employees with higher P-J fit will more easily be able to recognize issues, produce and suggest ideas, and implement them. Such an increase in innovative behavior may also occur for employees with lower P-J fit, who are motivated to make drastic changes to the current situation to correct the mismatch with their job; when they do not need to be afraid of their supervisor's negative reactions, they will be able to fully invest their remaining resources in such efforts, voicing ideas that can make changes in their job context and push them ahead, which may result in similar levels of innovative behavior to those of higher P-J fit. Overall, when abusive supervision is low, the relationship between P-J fit and innovative behavior may be strengthened, constituting a curve close to a symmetric U-shape. Therefore, the following hypotheses are proposed:

*Hypothesis 2: Abusive supervision will moderate the relationship between P-J fit and innovative behavior such that the non-linear (U-shaped) relationship between them is weakened when abusive supervision is high but strengthened when it is low*.

## 3 Methods

### 3.1 Participants

Data were collected from employee-supervisor dyads who work for several different organizations in South Korea to test the hypotheses empirically. The sample came from various organizations, such as banking, construction, or service organizations, and included both those public and private. Our sample selection criteria were general personnel, thus we excluded companies or divisions that were unlikely to be generalizable, such as hospitals. Similar to the snowballing sampling technique, we first personally contacted middle or senior managers of each organization and had them recommend employee-supervisor dyads suitable for the purpose of this study. For example, to ensure the correct evaluation of focal employees' innovative behaviors, employees and their immediate supervisors should interact with each other on a daily basis. The sampling approach is appropriate for this study because it is difficult to obtain randomized matched samples without the approval of HR departments.

### 3.2 Procedures

We had our questionnaires delivered to employees and their immediate supervisors via contact points, and respondents were instructed to seal and return their questionnaires via the same contact points. Only identification numbers appeared on the envelopes so that the respondents could be assured of anonymity. To alleviate concerns about common methods bias (Podsakoff et al., 2003), we asked employees to rate their P-J fit and perceived level of abusive supervision, while asking their immediate supervisors to evaluate the innovative behavior of focal employees.

After matching, the initial sample comprised 213 pairs of subordinate-leader dyads. After excluding cases with missing or unmatched data, 180 usable dyadic cases remained for the final analysis. Of employees, the average age was 39.02 years (SD = 9.64); 66.1% held a bachelor's degree, and most were full-time workers (95.0%). The average age of supervisors was 48.47 years (SD = 7.66), and also most (72.8%) held a bachelor's degree. Regarding gender distribution, the employee sample consists of 110 males (61.1%) and 70 females (38.9%). When matched with supervisors' genders, the same gender dyads were 129 (male employee-male leader 98, female employee and female leader 31) while mixed gender dyads were 51 (male employee-female leader 12, female employee and male leader 39). Table 1 provides the demographic characteristics of the respondents.

**Table 1:** Demographic characteristics of participants.

	**Employee**					**Supervisor**				
	**Frequency**	**Percentage (%)**	**Median**	**Range**	**Mode**	**Frequency**	**Percentage (%)**	**Median**	**Range**	**Mode**
**Age**			38.00	20–62	39			49	27–61	57
20–29	30		16.7			3	1.7			
30–39	73		40.6			28	15.6			
40–49	45		25.0			62	34.4			
50–59	31		17.2			84	46.7			
60–69	1		0.5			3	1.6			
**Gender**										
Male	110		61.1			137	76.1			
Female	70		38.9			43	23.9			
**Education level**										
High school	16		8.9			10	5.5			
Vocational college	23		12.8			19	10.6			
Bachelor degree	119		66.1			131	72.8			
Graduate school	21		11.7			20	11.1			
Others	1		0.5			0	0			
**Job type**										
Full-time	171		95.0							
Contract	4		2.2							
Temporary	1		0.6							
Part-time	1		0.6							
Others	3		1.6							
**Tenure with supervisor**			1.21		1					
< 1 year	61		33.9							
1 year−2 year	52		28.9							
2 year−5 year	43		23.9							
5 year <	24		13.3							

### 3.3 Measures

Established scales were used to measure variables. The English scales were translated into Korean following the conventional method of back translation (Brislin, 1980). Two bilingual academics independently translated and back translated the questionnaires. All items were measured on a seven-point Likert scale ranging from 1 (strongly disagree) to 7 (strongly agree). Before distributing the questionnaires to participants, we distributed them to several academics and potential respondents, and they gave us positive feedback that the questions were clear and easy to understand.

#### 3.3.1 Person-job fit (P-J fit)

Employees evaluated their person-job fit using Cable and DeRue's (2002) six-items, which combined the three-item needs-supplies fit and the three-item demands-abilities fit scale. The sample items are “There is a good fit between what my job offers me and what I am looking for in a job (needs-supplies fit)” and “The match is very good between the demands of my job and my personal skills (demands-abilities fit).”

#### 3.3.2 Abusive supervision

The same employees evaluated their supervisors' abusive supervision using Tepper (2000)'s 15-item abusive supervision scale. Sample items are “My supervisor ridicules me,” and “My supervisor tells me my thoughts or feelings are stupid.”

#### 3.3.3 Innovative behavior

The immediate supervisors evaluated the focal employees' innovative behaviors using six-item innovative behavior scale developed by Scott and Bruce (1994). The sample items are “This employee searches out new technologies, processes, techniques, and/or product ideas”, and “This employee promotes and champions ideas to others.”

#### 3.3.4 Control variables

Employees' age, gender, education, job type, tenure with the supervisor, and age, gender, and education of the supervisor were controlled. Previous research has found that these variables influence the main ones such as person-job fit, abusive supervision, and innovative behavior (e.g., Scott and Bruce, 1994; Leung et al., 2011; Zhang and Bednall, 2016; Liao et al., 2018; Watkins et al., 2019; Kim et al., 2022).

### 3.4 Analytical strategy

Prior to testing the hypothesized model, we first performed preliminary analyses, such as confirmatory factors analysis. In order to test hypotheses, we conducted hierarchical regression analyses. Before conducting the analysis, each independent variable and the moderating variable were mean-centered to alleviate potential multicollinearity issues (Aiken et al., 1991). A five-step procedure was conducted to test hypothesized relationships. First, eight control variables (employees' age, gender, education, job type, tenure with the supervisor, and age, gender, and education of the supervisor) were included in Step 1. In step 2, we put P-J fit to account for a potential linear pattern. In step 3, we entered a P-J fit squared to test hypothesis 1. Next, to test the moderating effect of abusive supervision on the relationship between P-J fit and innovative behavior, abusive supervision was put in step 4, followed by its interaction term with P-J fit and that with P-J fit squared.

## 4 Results

### 4.1 Preliminary analyses

Before testing the hypothesized model, a series of confirmatory factor analyses (CFAs) was conducted to ensure the constructs were distinct from the others. As shown in Table 2, the hypothesized three-factor model showed a good fit to the data (χ2 (24) = 38.37, *p* < 0.05, comparative fit index (CFI) = 0.99, Tucker–Lewis Index (TLI) = 0.99, and root mean square error of approximation (RMSEA) = 0.06) and provided a better fit than two- or one-factor models. Therefore, these results indicate that the three constructs are distinct from the others. The means, standard deviations, reliability, and correlations among the variables are shown in Table 3.

**Table 2:** Confirmatory factor analysis results.

**Model**	**χ2**	***df***	**CFI**	**TLI**	**RMSEA**	**Δχ2**	**Δ*df***
Hypothesized model: PJF^a^, AS^b^, INNOVB^c^	38.37	24	0.99	0.99	0.06		
Two-factor model: (PJF^a^ + AS^b)^, INNOVB^c^	135.91	26	0.93	0.91	0.15	97.54^**^	2
One-factor model: (PJF^a^, AS^b^, INNOVB^c)^	775.532	27	0.53	0.38	0.39	737.16^**^	3

The changes of Chi-square (Δχ2) and degree-of-freedom (Δdf) were against the hypothesized model.

PJF^a^, P-J fit; AS^b^, Abusive Supervision; INNOVB^c^, Innovative Behavior; CFI, Comparative Fit Index; TLI, Tucker Lewis Index; RMSEA, Root Mean Square of Error of Approximation.

^**^p < 0.01.

**Table 3:** Descriptive statistics and correlations.

**Variables**		**Mean**	**SD**	**1**	**2**	**3**
(1)	P-J fit	4.49	1.07	(0.91)		
(2)	Abusive Supervision	1.88	0.99	−0.18^*^	(0.97)	
(3)	Innovative Behavior	4.39	1.27	0.12	−0.20^**^	(0.97)

N = 180. Cronbach's alphas are on the diagonal in parentheses. ^*^p < 0.05; ^**^p < 0.01.

### 4.2 Hypothesis testing

**Figure 2:**
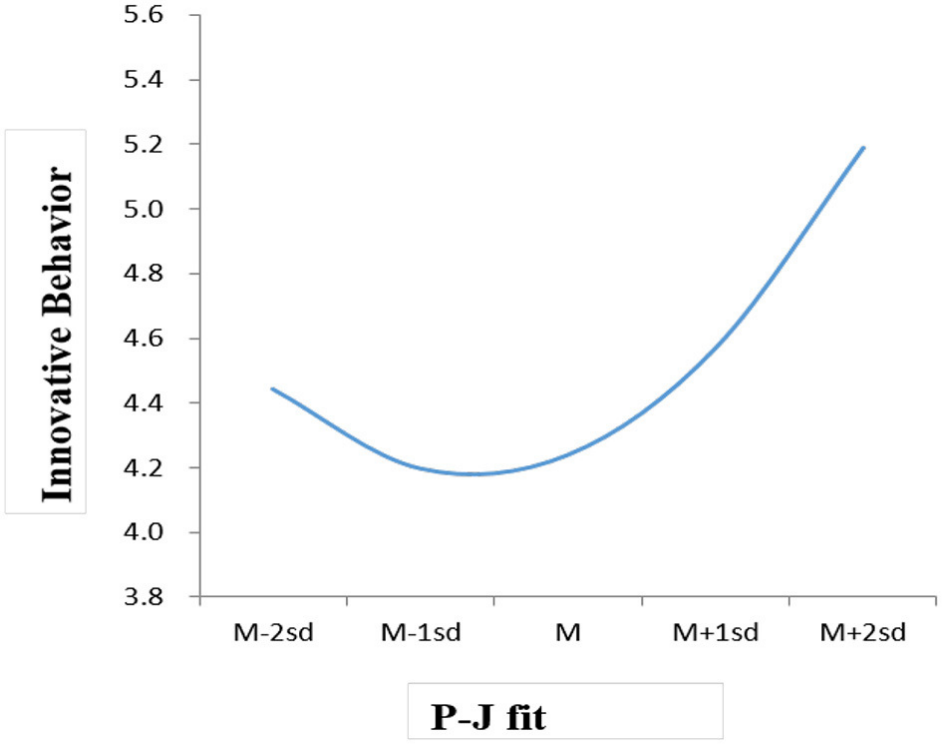
The non-linear effect of P-J fit on innovative behavior.

Table 4 presents the regression analysis results. Hypothesis 1 predicted that the relationship between P-J fit and innovative behavior would be non-linear. After controlling demographic variables, the relationship between P-J fit and innovative behaviors was not significant (β = 0.10, *n.s*). However, the coefficient for the P-J fit squared was significant (β = 0.15, *p* < 0.05; see **Model 3**). Thus, there is a non-linear relationship between P-J fit and innovative behavior. The relationship between the two is depicted following the recommendation of Cohen and Cohen (1983). As predicted, the results showed a non-linear relationship between P-J fit and innovative behavior (see Figure 2). Specifically, innovative behavior sharply declined until the P-J fit reached the lowest point of the average (from 2 SD to the lower limit of the mean), However, the innovative behavior began to increase once the P-J fit fell below the average (from −1SD to −2 SD), resulting in a concave curve. Therefore, Hypothesis 1 was supported.

**Table 4:** Hierarchical regression results for hypothesized relationships.

	**Innovative behavior**									
	**Model 1**		**Model 2**		**Model 3**		**Model 4**		**Model 5**	
	β	***t***	β	***t***	β	***t***	β	***t***	β	***t***
Employee's age	0.04	0.46	0.02	0.19	0.01	0.10	0.01	0.10	0.01	0.07
Employee's gender	−0.11	−1.43	−0.12	−1.53	−0.11	−1.40	−0.12	−1.59	−0.12	−1.55
Employee's education level	0.10	1.35	0.11	1.47	0.11	1.51	0.11	1.52	0.10	1.38
Employee's job type	−0.22^**^	−3.16	−0.23^**^	−3.22	−0.23^**^	−3.31	−0.22^**^	−3.11	−0.18^*^	−2.34
Employee's tenure with supervisor	0.00	−0.03	0.01	0.11	0.01	0.15	0.02	0.26	0.03	0.46
Supervisor's age	0.32^***^	4.08	0.31^***^	3.95	0.30^***^	3.95	0.28^***^	3.61	0.27^**^	3.54
Supervisor's gender	0.07	0.30	0.09	1.16	0.10	1.28	0.07	0.94	0.07	0.88
Supervisor's education level	0.02	0.95	0.02	0.28	0.04	0.50	0.01	0.19	0.00	0.04
P-J fit			0.10	1.40	0.15	1.92	0.12	1.54	0.11	1.39
P-J fit squared					0.15^*^	2.07	0.15^*^	2.03	0.18^*^	2.46
Abusive supervision							−0.14	−1.83	0.03	0.26
P-J fit X abusive supervision									0.07	1.01
P-J fit squared X abusive supervision									−0.23^*^	−2.26
*R^2^*	0.13		0.13		0.15		0.16		0.18	
*Δ R^2^*			0.01		0.02		0.02		0.03	
*F*	4.30^***^		4.06^***^		4.16^***^		4.14^***^		4.11^***^	

N = 180. ^*^Entries are standardized regression coefficients. ^*^p < 0.05; ^**^p < 0.01; ^***^p < 0.001 (two-tailed).

**Figure 3:**
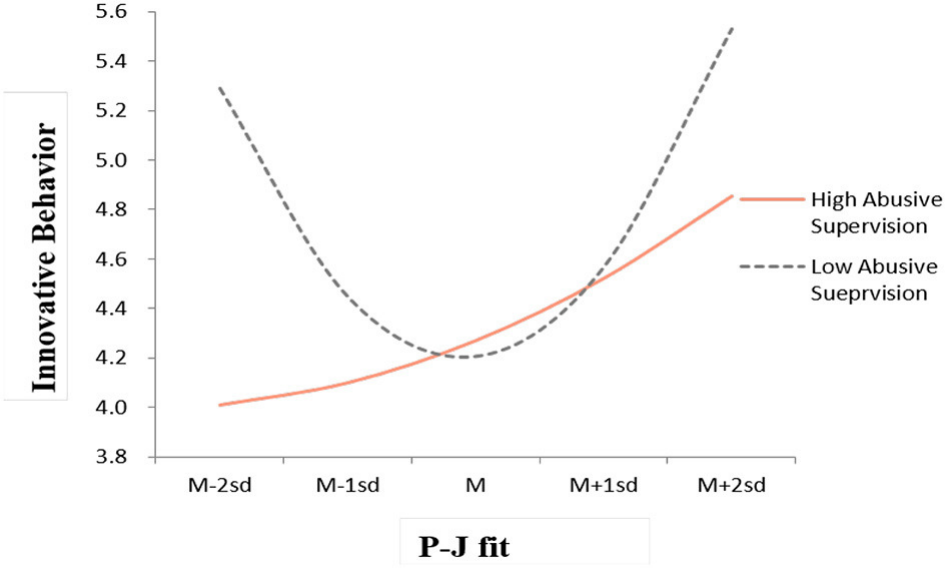
The moderating effect of abusive supervision on the relationship between P-J fit and innovative behavior.

Hypothesis 2 predicted that the relationship between P-J fit and innovative behavior would be weakened when abusive supervision was high and strengthened when it was low. The interaction between the P-J fit squared and abusive supervision was significant (β = −0.23, *p* < 0.05; see **Model 5**). Figure 3 illustrates the moderating effects of abusive supervision. At high levels of abusive supervision, the pattern of the relationship between P-J fit and innovative behavior was weakened overall, showing a linear rather than curvilinear pattern, and the decline in innovative behavior tended to be deeper below the average of the P-J fit (from the mean to −2SD) than beyond the average of the P-J fit (from the mean to 2SD). Consequently, the pattern exhibited a positive linear shape. In contrast, when abusive supervision was low, the U-shaped relationship between person-job fit and innovative behavior was strengthened, showing clarity in its shape. Specifically, the levels of innovative behavior were similarly high at both ends of the P-J fit, thereby resulting in a symmetrical U-shaped curve. Following the procedure suggested by Aiken et al. (1991), we also conducted a series of simple slope tests. The examination of the simple slope revealed that, for employees under the condition of low abusive supervision, the slope is negative and significant at the lower level of P-J fit (−2SD: *b* = −0.90, *t* = −2.95, *p* < 0.01) while being positive and significant at the higher level of P-J fit (2SD: *b* = 1.00, *t* = 3.08, *p* < 0.01). For those under the condition of high abusive supervision, the slopes were positive and non-significant both at the lower level of P-J fit (- 2SD: *b* = 0.04, *t* = 0.15, *n.s*.) and at the higher level of (2SD: *b* = 0.30, *t* = 0.94, *n.s*.). Thus, Hypothesis 2 was supported.

## 5 Discussion

It has been perceived that P-J fit leads to positive organizational outcomes, such as innovative behavior. Acknowledging some inconsistent findings from previous research, this study tested the nonlinear assumption of the relationship between P-J fit and innovative behavior. The study results show that P-J fit has a non-linear relationship with innovative behavior. When P-J fit initially diminished, employees' innovative behavior also diminished. However, once the P-J fit reached the threshold, the levels of innovative behavior rather increased. Furthermore, at a high level of abusive supervision, the relationship was significantly weakened, showing an almost positive linear pattern. Conversely, at low levels of abusive supervision, the non-linear relationship was strengthened, constituting a symmetric U-shaped curve.

### 5.1 Theoretical implications

The present study adds to the discipline of applied psychology mainly in three ways.

First, by suggesting that the relationship between P-J fit and employee innovative behavior is not a simple linear but can be a curvilinear, this present finding points out that, although the importance of “good fit” between person and job cannot be overstated, “poor fit” should not always be considered “no-good” because the deficit in the fit can rather bring about the similar level of innovative performance. This corroborates Luis et al.'s (2020) explanation that stress can evoke innovative behavior as a response to the challenges of self-growth and self-transformation, which sheds light on individuals' proactive efforts to change their environments. Thus, our finding of the non-linear relationship offers a more sophisticated understanding of the outcomes of job fit. However, it should also be noted that the lack of P-J fit may rather be harmful to other work outcomes. For example, Chi et al. (2020) found that newcomers' person-job misfits (N-S and D-A misfits) were found to be positively related to actual turnover and negatively related to task performance. Similarly, Khan et al. (2022) reported that job over qualification led to counterproductive behavior via job boredom. Therefore, the relationship between low P-J fit and innovative behavior needs to be considered, along with other work outcomes.

Second, while the present study's results provide evidence for the non-linear relationship between P-J fit and innovative behavior, the results contrast with some research results suggesting the inverted U-shaped relationship between stressors and creative or innovative performance. For example, Wang (2022) reported that dissatisfaction with the status quo has an inverted U-shaped relationship with innovative behavior. Similarly, Montani et al. (2020) suggested that work overload has an indirect inverted U-shaped relationship with innovative work behavior via work engagement. In contrast, our findings correspond more with the study results of Nygaard and Dahlstrom (2002) and Leung et al. (2011), which showed the U-shaped relationship between the role stress and (innovative) performance based on the triphasic model (Selye, 1950, 1974). Nonetheless, with the inconsistency in mind, we agree with the conclusion of Byron et al.'s (2010) meta-analysis that the effects of stressors on creative performance depend on their level and type. As such, the present study suggests that the relationship between P-J fit and innovative behavior needs a more sophisticated understanding.

Third, by demonstrating that the relationship between P-J fit and innovative behavior can differ significantly depending on the influence of abusive supervision, the present study adds nuances to our understanding of the relationship between P-J fit and innovative behavior. The pattern constituted a linear pattern in the condition of high abusive supervision and a strongly U-shaped pattern in the condition of low abusive supervision. It is in line with the research suggesting that moderating variables can determine whether the relationships between work outcomes and their predictors are curvilinear or linear (e.g., Guo et al., 2020; Yue and Huang, 2024). Thus, we note that the conflicting insights from previous studies might, in part, stem from the complex interplay with surrounding factors. Therefore, this study highlights the importance of considering contextual factors in understanding employee responses to job fit discrepancies.

Fourth, the present study integrates multiple theoretical perspectives to understand individuals' coping responses: the triphasic model of stress and conservation of resources theory. In line with the two theoretical perspectives, the paradoxical increase in innovative behavior of employees with low P-J fit indicates that individual employees are not merely passive reactors to stressors such as job misfit but are also active agents who take the initiative of innovative changes if needed (Hobfoll et al., 2018). On the other hand, in the presence of abusive supervision, the drastic decline in the innovative behavior of employees with lower P-J fit also supports the notion that too much stress or loss may lead individuals to cease their coping efforts (Selye, 1950, 1974; Hobfoll et al., 2018). Thus, the Present study suggests how multiple theories can connect at their intersection to explain psychological reactions and behavioral outcomes within organizational settings. However, it should also be noted that locus of control, the extent to which an individual believes that he or she has control over one's life (Ng et al., 2006), may play a core role in the non-linear relationship between P-J fit and innovative behavior. The more an individual believes he or she can control his or her situation, the more innovative the person may be in the face of job fit deficiency. Thus, future research testing the role of locus control in the relationship between P-J fit and innovative behavior may yield interesting results.

Finally, unlike the similar level of innovative behaviors of both employees with higher and lower P-J fit in the absence or low abusive supervision, a stark contrast between the innovative behavior of employees with a higher P-J fit and that of the lower P-J fit in high abusive supervision also underscores the significance of P-J fit as a key personal resource (Wheeler and Halbesleben, 2009) that can mitigate the detrimental impacts of adverse work conditions.

### 5.2 Practical implications

The findings of this study have practical implications for organizations seeking to promote innovative behavior among employees.

First, organizations should recognize that not only employees with high P-J fit but also those with low P-J fit may engage in innovative behavior as a struggle to narrow the gaps in fit. Although the importance of person-job fit in recruiting and placement cannot be overstated, in reality, not all employees are allocated jobs that match them perfectly. Thus, by recognizing the nuanced nature of the relationship between P-J fit and innovative behavior, organizations can tailor their interventions to create work environments that support employees in enhancing their capacity to enact innovative changes. For example, organizations can promote open communication systems in which employees can express their ideas more freely to improve their job performance and provide funds and delegation so that they can take the initiative to realize innovative ideas. Such systemically supported resources will help prevent the decline in innovative behaviors of those with less than perfect P-J fit while fueling the initiative actions of those with low P-J fit.

Second, the results of the present study still support the wisdom that the perception of good P-J fit can be a valuable source of innovative behaviors. The findings of the present study show that even in adverse situations, the decline in innovative behavior was relatively gentle for employees with a higher P-J fit. Thus, organizations can cultivate innovative behaviors among employees by thoroughly considering the fit between candidates' attributes and job requirements during the recruitment and selection processes. In addition, designing jobs that align with incumbents' skills, knowledge, abilities, and values can promote a sense of fit and thus encourage innovative behavior.

Third, although the detrimental effects of abusive supervision are widely known, the results of this study show that employees with low P-J fit may be particularly vulnerable to its negative impacts and may even adopt defensive postures to protect themselves, resulting in very low levels of innovative behavior. Thus, this study underscores the importance of addressing abusive supervision as a potential barrier to innovative behavior. As such, promoting innovative behavior among employees requires combined efforts: facilitating P-J fit and eradicating abusive supervision.

### 5.3 Limitations and future research

First, while the present study suggests that employees with low P-J fit can show a high level of innovative behavior, it used a cross-sectional research design and did not trace how their innovative behavior changes over time. However, those with a low fit may lack the skills and knowledge needed to realize novel ideas (Cable and DeRue, 2002). Thus, it is possible that they easily lead to higher burnout or psychological strains, as increasing innovative behavior may be emotionally and cognitively demanding. Furthermore, the probability that their efforts will fail may be higher than that of those with high P-J fit, which may lead to voluntary or involuntary turnover in the long term. Thus, future research using a longitudinal design to track the long-term effects of P-J fit on employees' innovative behaviors will advance our understanding of its curvilinear effects. For example, recent research used a three-wave design over a year to test how the changes in P-J fit influenced employees' job outcomes (Kim et al., 2020).

Second, the present study was conducted in South Korea, which has high power distance and performance-oriented culture. Although this cultural context provides a good research environment to test the hypotheses of this study, it may also function as a limitation in that the results may less be generalizable to some country settings. For example, employees in countries with low-performance orientation cultures, such as Russia (House, 2001), may not drastically increase their innovative performance despite the widened gap between desired and unsatisfactory P-J fit. Also, it is plausible that employees in countries with low power distance cultures, such as the U.S. (Mead and Andrews, 2009), may less sensitively react to abusive supervision, and subsequently, the declines in their innovative performance may less be deep even at a low level of P-J fit than those of employees in high power distance settings. In such a case, the moderating effect of abusive supervision on the relationship between P-J fit and innovative behavior will still likely be close to U-shaped rather than linear. Thus, research replicated in other cultural settings may also provide meaningful insights into those relationships.

The third limitation of the present study lies in its sample and analysis issues. Our sample, comprised of 180 supervisor-employee dyads, is relatively small. Thus, future research using a bigger sample will yield more robust results. Also, we could not use the organizational type as a control variable despite our sample including both public and private organizations. This is because, while ensuring respondents about the confidentiality and anonymity of the research, we did not stringently trace their identifiable information except those for the research purpose. However, it is plausible that the lack of P-J fit may affect employees in public and private organizations differently due to different levels of performance pressure. Relatedly, one may be concerned about the potential multilevel issue because certain employees and supervisors are nested within organizations. Although we believe these issues may be alleviated in that data comes from various organizations, nonetheless, it may be possible for future research taking into account the organizational type or a multilevel modeling approach to bring about different implications from the present study. In addition, future research may consider using recently introduced analysis techniques, such as the two-line test (Simonsohn, 2018) or extensions of the Johnson-Neyman technique (Miller et al., 2013), to further explore the curvilinear and moderated relationships.

## 6 Conclusion

The present study hypothesized and tested the non-linear assumption of the relationship between P-J fit and innovative behavior. The results of the present study indicate that not only individual employees with a higher P-J fit but also those with a lower fit can engage in innovative behaviors to resolve the lack of job fit. Furthermore, the present study shows that the pattern of the relationship between P-J fit and innovative behavior may be positively linear at a high level of abusive supervision, whereas symmetric U-shaped at a low level of abusive supervision. As such, the present study sheds light on individuals' proactive efforts to cope with the lack of job fit as human agents while confirming the importance of P-J fit as an important resource to offset the detrimental impacts of abusive supervision.

## Data Availability

The raw data supporting the conclusions of this article will be made available by the authors, without undue reservation.

## Appendix

**Person-Job fit** (P-J fit) (α= 0.91)

1. There is a good fit between what my job offers me and what I am looking for in a job.

2. The attributes that I look for in a job are fulfilled very well by my present job.

3. The job that I currently hold gives me just about everything that I want from a job.

4. The match is very good between the demands of my job and my personal skills.

5. My abilities and training are a good fit with the requirements of my job.

6. My personal abilities and education provide a good match with the demands that my job places on me.

**Innovative Behavior** (α= 0.97)

1. This employee searches out new technologies, processes, techniques, and/or product ideas.

2. This employee generates creative ideas.

3. This employee promotes and champions ideas to others.

4. This employee investigates and secures funds needed to implement new ideas.

5. This employee develops adequate plans and schedules for the implementation of new ideas.

**Abusive Supervision** (α= 0.97)

1. My supervisor ridicules me.

2. My supervisor tells me my thoughts or feelings are stupid.

3. My supervisor gives me the silent treatment.

4. My supervisor puts me down in front of others.

5. My supervisor invades my privacy.

6. My supervisor reminds me of my past mistakes and failures.

7. My supervisor doesn't give me credit for jobs requiring a lot of effort.

8. My supervisor blames me to save himself/herself embarrassment.

9. My supervisor breaks promises he/she makes.

10. My supervisor expresses anger at me when he/she is mad for another reason.

11. My supervisor makes negative comments about me to others.

12. My supervisor is rude to me.

13. My supervisor does not allow me to interact with my coworkers.

14. My supervisor tells me I'm incompetent.

15. My supervisor lies to me.
